# Optimal Organization of Functional Connectivity Networks for Segregation and Integration With Large-Scale Critical Dynamics in Human Brains

**DOI:** 10.3389/fncom.2021.641335

**Published:** 2021-03-31

**Authors:** Xinchun Zhou, Ningning Ma, Benseng Song, Zhixi Wu, Guangyao Liu, Liwei Liu, Lianchun Yu, Jianfeng Feng

**Affiliations:** ^1^Key Laboratory of Theoretical Physics of Gansu Province, Lanzhou Center for Theoretical Physics, Lanzhou University, Lanzhou, China; ^2^School of Mathematical Sciences and Centre for Computational Systems Biology, Fudan University, Shanghai, China; ^3^Department of Magnetic Resonance, Lanzhou University Second Hospital, Lanzhou, China; ^4^College of Electrical Engineering, Northwest University for Nationalities, Lanzhou, China; ^5^School of Mathematical Sciences, School of Life Science and the Collaborative Innovation Center for Brain Science, Fudan University, Shanghai, China

**Keywords:** fMRI, functional connection networks, criticality, DTI, Greenberg-Hasting model, E/I ratio

## Abstract

The optimal organization for functional segregation and integration in brain is made evident by the “small-world” feature of functional connectivity (FC) networks and is further supported by the loss of this feature that has been described in many types of brain disease. However, it remains unknown how such optimally organized FC networks arise from the brain's structural constrains. On the other hand, an emerging literature suggests that brain function may be supported by critical neural dynamics, which is believed to facilitate information processing in brain. Though previous investigations have shown that the critical dynamics plays an important role in understanding the relation between whole brain structural connectivity and functional connectivity, it is not clear if the critical dynamics could be responsible for the optimal FC network configuration in human brains. Here, we show that the long-range temporal correlations (LRTCs) in the resting state fMRI blood-oxygen-level-dependent (BOLD) signals are significantly correlated with the topological matrices of the FC brain network. Using structure-dynamics-function modeling approach that incorporates diffusion tensor imaging (DTI) data and simple cellular automata dynamics, we showed that the critical dynamics could optimize the whole brain FC network organization by, e.g., maximizing the clustering coefficient while minimizing the characteristic path length. We also demonstrated with a more detailed excitation-inhibition neuronal network model that loss of local excitation-inhibition (*E*/*I*) balance causes failure of critical dynamics, therefore disrupting the optimal FC network organization. The results highlighted the crucial role of the critical dynamics in forming an optimal organization of FC networks in the brain and have potential application to the understanding and modeling of abnormal FC configurations in neuropsychiatric disorders.

## Introduction

Functional connectivity (FC) analysis of resting state human brain allows to understand how the functional networks are organized, how this organization is related to behavior, and how it changes in case of pathology (van den Heuvel and Hulshoff Pol, 2010; Lee et al., 2013; Yu et al., 2016). Recent studies have identified the so-called resting-state networks which consist of anatomically separated, but functionally linked brain regions that show a high level of ongoing FC during rest (Heine et al., 2012; Raichle, 2015). The graph theoretical analysis of resting-state functional magnetic resonance imaging (fMRI) has revealed the “small-world” feature of the whole brain functional connectivity network (Rubinov and Sporns, 2010). Compared with random networks, small-world networks exhibit shorter characteristic path length but higher clustering coefficients (Watts and Strogatz, 1998). This specific organization of functional network is believed to benefit the higher-level cognitive functions requiring the integration of information from different brain regions (Watts and Strogatz, 1998), maximize efficiency at a minimal cost for effective information processing between multiple brain regions (Achard and Bullmore, 2007), and promote low wiring costs (Bassett and Bullmore, 2006). The small-world organization of brain functional network is likely to be related to human intellectual performance (van den Heuvel et al., 2009) and disrupted with normal aging (Wang et al., 2010). Extensive studies also showed that this small-world properties of functional network are altered by diseases such as schizophrenia (Liu et al., 2008), AD (Sanz-Arigita et al., 2010), autism (Rudie et al., 2013), etc. Specifically, the alterations are normally characterized by increased characteristic path length, as well as decreased clustering coefficient and efficiency [for an example, pleases see Ref. (Liu et al., 2008) for details], implying the disrupted organization of FC networks for integration and segregation. However, little is known about the underlying dynamics based on which this optimal FC network is established, and how its disruption induced by disease is associated with changes in brain dynamics.

The theory from statistical physics has predicted that resting state brain dynamics operates close to a critical point, hallmarked by power-law distributions of spatiotemporal cascades of activity-termed neuronal avalanche. Scale-free avalanches have been observed in different scales of neural systems with different methods (Beggs and Plenz, 2003; Gireesh and Plenz, 2008; Ribeiro et al., 2010), including local field potentials (Thiagarajan et al., 2010; Plenz, 2012), human electroencephalography (EEG) (Meisel et al., 2013), magnetoencephalogram (MEG) (Palva et al., 2013; Shriki et al., 2013), and fMRI (He, 2011; Tagliazucchi et al., 2012). It is suggested that there are many computational advantages for the neural systems being poised around this critical point. It maximize the number of meta-stable states (Haldeman and Beggs, 2005), the dynamic range to the input stimuli (Shew et al., 2009; Gautam et al., 2015), as well as the information capacity and transmission (Shew et al., 2011) of the cortical neural networks. Furthermore, cortical EI balance are found to be crucial for the forming of critical behavior at multiple levels of neuronal organization (Poil et al., 2012), perhaps achieved through self-organization with synaptic plasticity (Stepp et al., 2015). On the other hand, a leading theory, proposed over a decade ago as a model for autism (Rubenstein and Merzenich, 2003), holds that brain disorders arise from imbalanced EI in brain circuitry. This concept has since been applied to many other brain disorders, such as schizophrenia, tuberous sclerosis, and Angelman syndrome (O'Donnell et al., 2017). These studies have led to the conjecture that criticality may be a signature of healthy neural systems, and conversely excursion from such an optimal point may be responsible for a diversity of brain disorder (Massobrio et al., 2015; Cocchi et al., 2017).

Recent modeling studies have also revealed crucial role of critical dynamics in understanding the relation between large scale brain architecture and function. For example, the spatial organization of resting state networks observed in the resting state fMRI data, such as default mode network, emerge at the critical point in the dynamic network derived from human brain neuroanatomical connections (Haimovici et al., 2013). The structure-function coupling is maximal when the global network dynamics operate at a critical point (Deco et al., 2014a), and the decoupling of functional connectivity from anatomical constraints is found in the brains losing consciousness, accompanied with fading signature of criticality (Tagliazucchi et al., 2016). In addition, the local excitation/inhibition ratio (*E*/*I* ratio) significantly improves the model's prediction of the empirical human functional connectivity at the large-scale brain level (Deco et al., 2014b). The loss of small-world organization of FC networks and failure of critical dynamics in diseased brain implies the potential relationship between them. However, it is still not clear how the organization of the FC network depends on the large-scale critical dynamics in brains.

In this work, we answered this question by investigating: (i) the correlation between topological metrics of FC network and the long-range temporal correlations (LRTCs) of BOLD signals in fMRI data of healthy subjects; (ii) the dependence of these metrics on the control parameter (excitation threshold) in a toy model which combines the structural diffusion tensor imaging (DTI) and Greenberg-Hasting (GH) dynamics around the critical point; (iii) the impact of local *E*/*I* ratio on the critical dynamics and thus the functional network metrics in a biological plausible whole brain model. We showed that with the critical dynamics, the brain FC network exhibited optimized organization, characterized by maximized efficiency and clustering coefficient, but shortest characteristic path length. We also showed that local *E*/*I* ratio have a great influence on this large-scale critical dynamics and organization of FC networks. We discussed the potential application of our findings to the understanding and modeling of abnormal FC configurations in brain disorders.

## Results

### Correlation of Network Metrics With LRTCs in Resting-State fMRI Data

We first assessed LRTCs in BOLD signals from the resting-state fMRI data of 95 healthy subjects by computing the Hurst exponent in the temporal domain using classical rescaled range (RS) method (Blythe and Nikulin, 2017) (“**METHOD AND MATERIALS**,” “**Hurst Exponent H**”). A Hurst exponent in the range 0.5 < *H* < 1 indicates the presence of LRTCs, i.e., a high value in the series will probably be followed by another high value. An exponent of 0 < *H* < 0.5 is obtained when the time series is anticorrelated (switching between high and low values in consecutive time steps). The uncorrelated temporal activity with exponential decay of the autocorrelation function yields an exponent of *H* = 0.5. After preprocessing with the standard preprocessing procedure (“**METHOD AND MATERIALS**,” “**fMRI Data Acquisition and Preprocessing**”), the automated anatomical labeling atlas (AAL) (Tzourio-Mazoyer et al., 2002) was used to parcellate the brain into 90 brain regions, and the mean BOLD signals were extracted in each brain region by averaging the signals of all voxels within the region. For each subject, the Hurst exponents were calculated for each mean BOLD time series, and the mean Hurst exponent (*H*) from 90 brain regions was taken as a measure of LRTCs at the whole brain level for this subject. The Hurst exponent reflects the temporal correlations of a signal. The group averaged Hurst exponent in our study is 0.8628 ± 0.0188, indicating the long-range memory of the BOLD signals in human brain (He, 2011). However, the variance in the Hurst exponent among subjects should not be ascribed to noise sources, such as physiological noise. On the contrary, considering criticality as a theory of long-range fluctuation in the human brain, it reflects the different intrinsic brain dynamics among subjects that can be described by a departure from the criticality (Blythe and Nikulin, 2017), as we demonstrated below.

**Figure 1:**
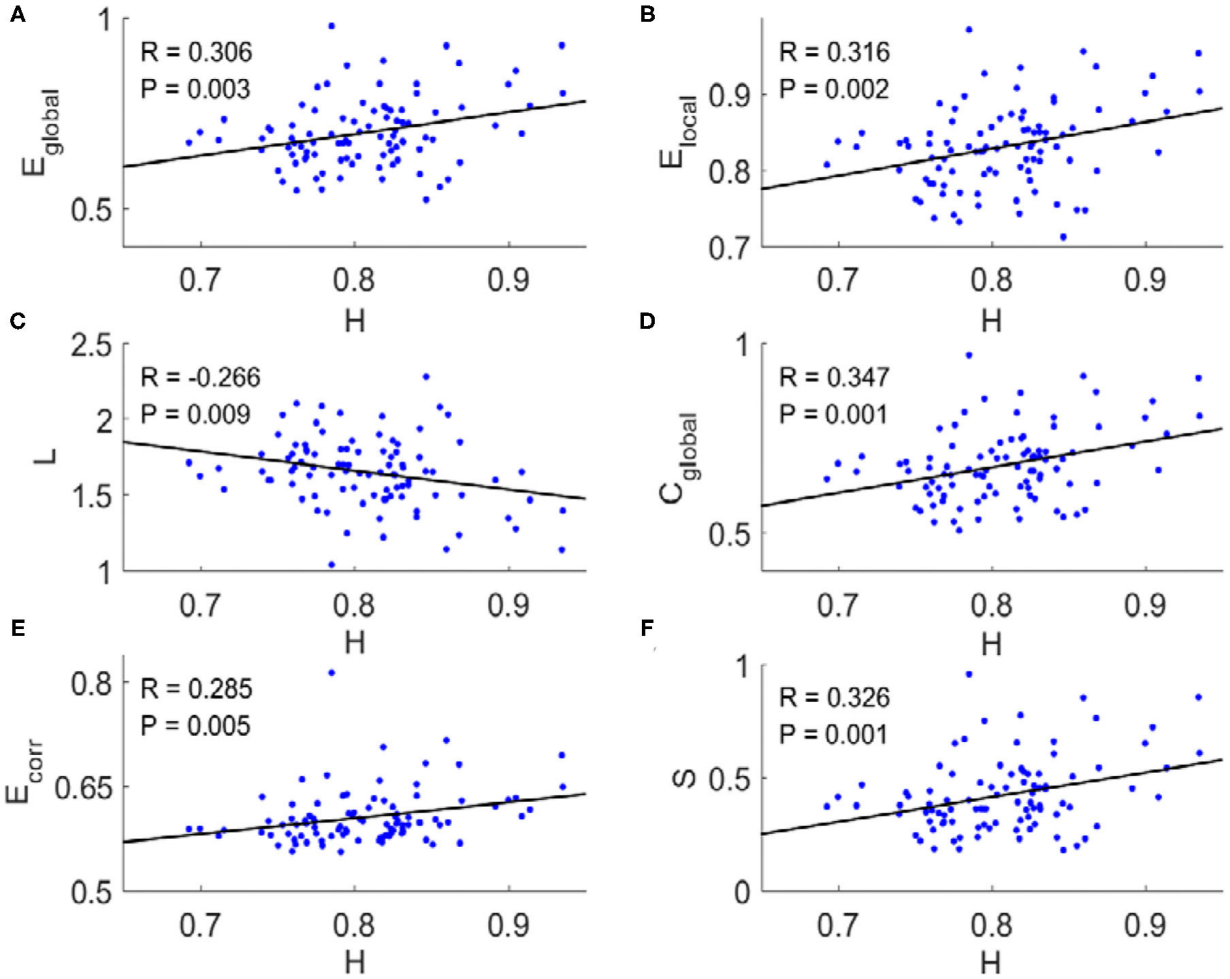
Scatter plots with trend line showing the dependence of topological metrics of FC network on Hurst exponents. **(A)** Global efficiency. **(B)** Local efficiency. **(C)** Characteristic path length. **(D)** Clustering coefficient. **(E)** Mean connection strength. **(F)** Sparsity. The topological metrics of FC networks were calculated with threshold *T*_*d*_ = 0.4. Pearson correlation coefficient (*R*) for all six topological metrics were significant (*p* < 0.01).

For each subject, we applied a binarizing threshold *T*_*d*_ to the absolute values of the correlation coefficients among mean BOLD signals from 90 brain regions to construct the FC network. Then six typical topological metrics, namely global and local efficiency (*E*_global_ and *E*_local_), characteristic path length (*L*), clustering coefficient (*C*_global_), mean connection strength (*E*_corr_), and sparsity (*S*) of the FC networks for each subject were calculated (“**METHOD AND MATERIALS**,” “**Network Metrics**”). We found there existed significant correlations between these metrics and the Hurst exponents (Figure 1). The longer temporal memory in BOLD signals yields higher global efficiency (Figure 1A), local efficiency (Figure 1B), clustering coefficient (Figure 1D), mean connection strength (Figure 1E), and sparsity (Figure 1F), but shortest characteristic path length (Figure 1C).

**Figure 2:**
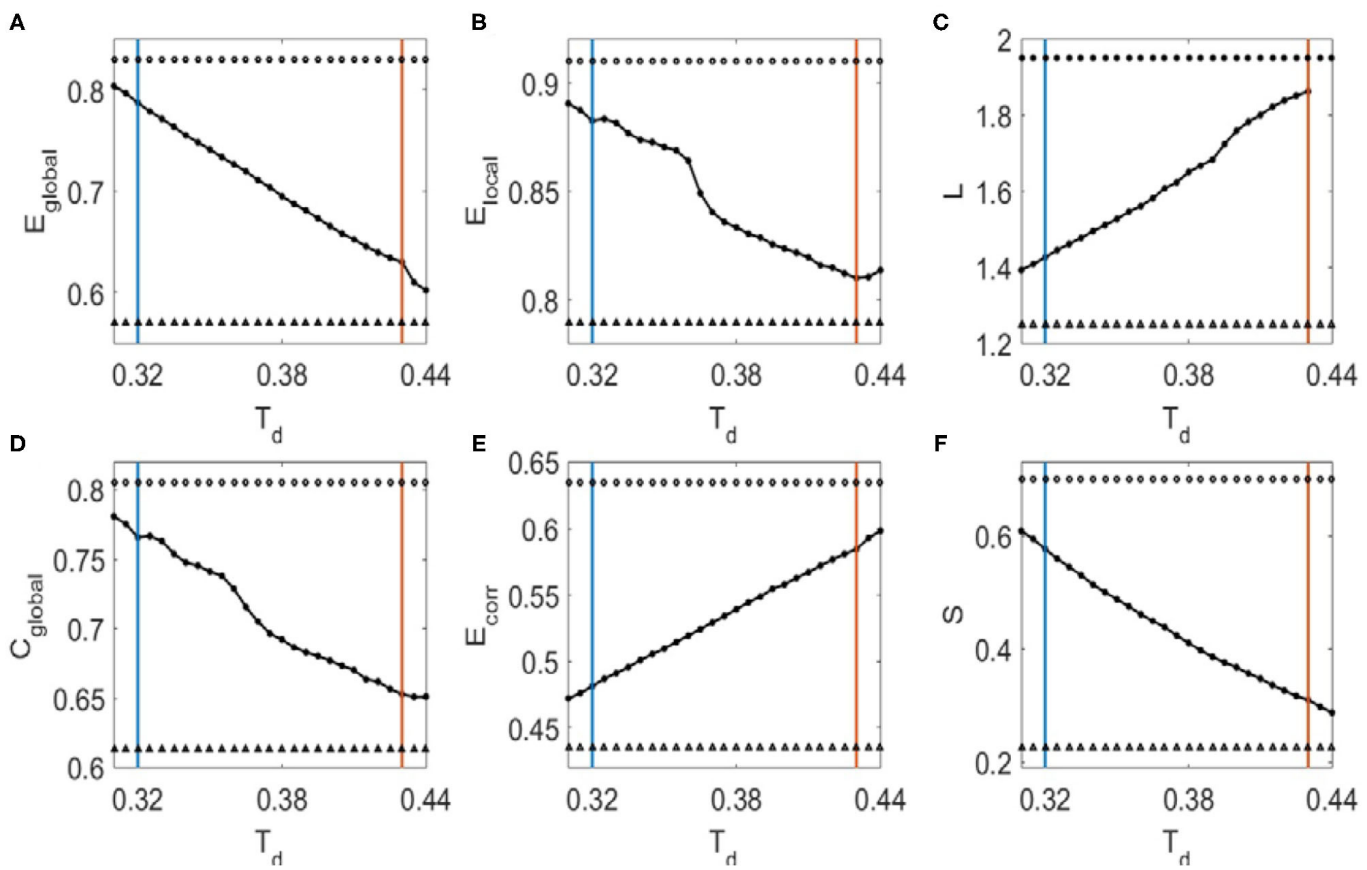
The dependence of topological metrics of the FC networks and their correlations with Hurst exponents on the threshold *T*_*d*_. **(A)** Global efficiency. **(B)** Local efficiency. **(C)** Characteristic path length. **(D)** Clustering coefficient. **(E)** Mean connection strength. **(F)** Sparsity. The red vertical lines mark the upper criteria above which there is no isolated node in the network, and the blue vertical lines mark the lower criteria below which the global efficiency curve for the brain networks is less than the global efficiency curve for the random networks. Open circles indicate that the correlation coefficients between the Hurst exponent and the corresponding topological metrics are larger than 0.26, where filled circles mark the correlation coefficients that is smaller than −0.26. Triangles mark the corresponding *p*-values of the correlation analysis that is significant (*p* < 0.01).

We then investigated the dependence of topological metrics and their correlations with Hurst exponents on the binarized threshold *T*_*d*_. We first determined the small-world regime of the FC networks for *T*_*d*_ (Liu et al., 2008). The upper criteria for *T*_*d*_ are so set to make sure there is no isolated node in the network (red vertical lines in Figure 2). To determine the lower criteria for *T*_*d*_, we compared the global efficiency of brain FC networks with that estimated in a random graph with the same degree distribution over a range of network sparsity (Supplementary Figure 1A). Then the lower criteria are set as the smallest value of the threshold *T*_*d*_ (blue vertical line in Figure 2) with which the global efficiency curve for the brain networks is below the global efficiency curve for the random networks. In this range of threshold *T*_*d*_, the Hurst exponents and the topological metrics are significantly correlated (Figure 2, the threshold values with correlation coefficients *R* > 0.26 are marked with open circles and *R* < −0.26 with filled circles. The corresponding *p*-values are indicated with triangles if *p* < 0.01). It was also noticed in Figure 2 that as the threshold *T*_*d*_ increases, the global efficiency (Figure 2A), local efficiency (Figure 2B), clustering coefficient (Figure 2D), and sparsity (Figure 2F) decrease, whereas the characteristic path length (Figure 2C) and the mean connection strength (Figure 2E) increase. The binarizing threshold dependent changes of these topological metrics are in line with previous study, e.g., Ref. (Liu et al., 2008).

### The Critical Dynamics in the DTI+GH Brain Network Model

**Figure 3:**
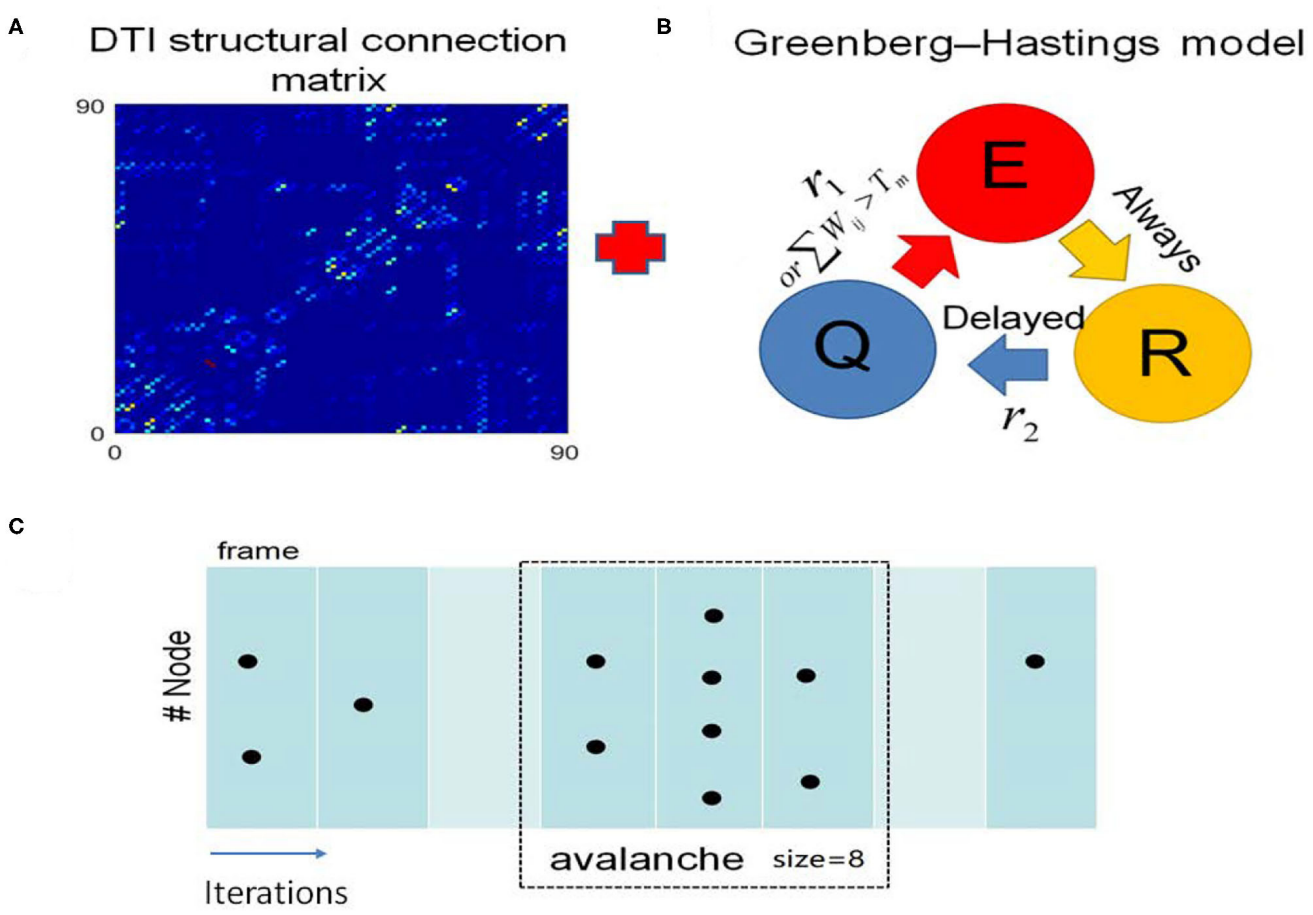
The DTI+GH whole brain model. **(A)** The DTI structural connection matrix. **(B)** The Greenberg-Hastings (GH) cellular automaton model for dynamics of each brain region. The GH model has three states: quiescent (*Q*), excitation (*E*), and refractory (*R*). The colored arrows indicated the transition between these states. The transition from *Q* to *E* can happen with a small probability *r*_1_, or if the sum of the connection weights *w*_*ij*_ with the active neighbors *j* is higher than a threshold *T*_*m*_. Once the system is excited, it always goes to *R*. Then it transits from *R* to *Q* with a probability *r*_2_ after several steps of delaying. **(C)** Demonstration of the method to extract avalanches from simulation of the whole brain model. The spatial activity in several simulation step is assigned as a frame (consecutive frames are divided by white lines). An avalanche is defined as the consecutive frames that are preceded by a blank frame (in which no activation occurs, marked with light cyan) and ended by a blank frame. The avalanche size is the total number of excited nodes in this avalanche. Black dots represent the excited nodes that are in the state *E*.

We built a toy brain network model which combines the DTI structural connection data among the 90 brain regions and GH excitable cellular automatons to simulate the BOLD signals from 90 brain regions (Figure 3, see details in the “**METHOD AND MATERIALS**,” “**DTI+GH Brain Network Model**”). In this model, the DTI connection data provides the number of fibers connecting every two brain regions, which is taken as the connection weights among the regions. The regional dynamics is given by simple rules that describe the excitation of the active media. Previous work has demonstrated that such simple dynamical brain model is sufficient to replicate fundamental features of spontaneous brain activity observed in fMRI data. For example, the resting state networks, such as default mode network, emerge in such kind of whole brain models with the critical dynamics (Haimovici et al., 2013).

**Figure 4:**
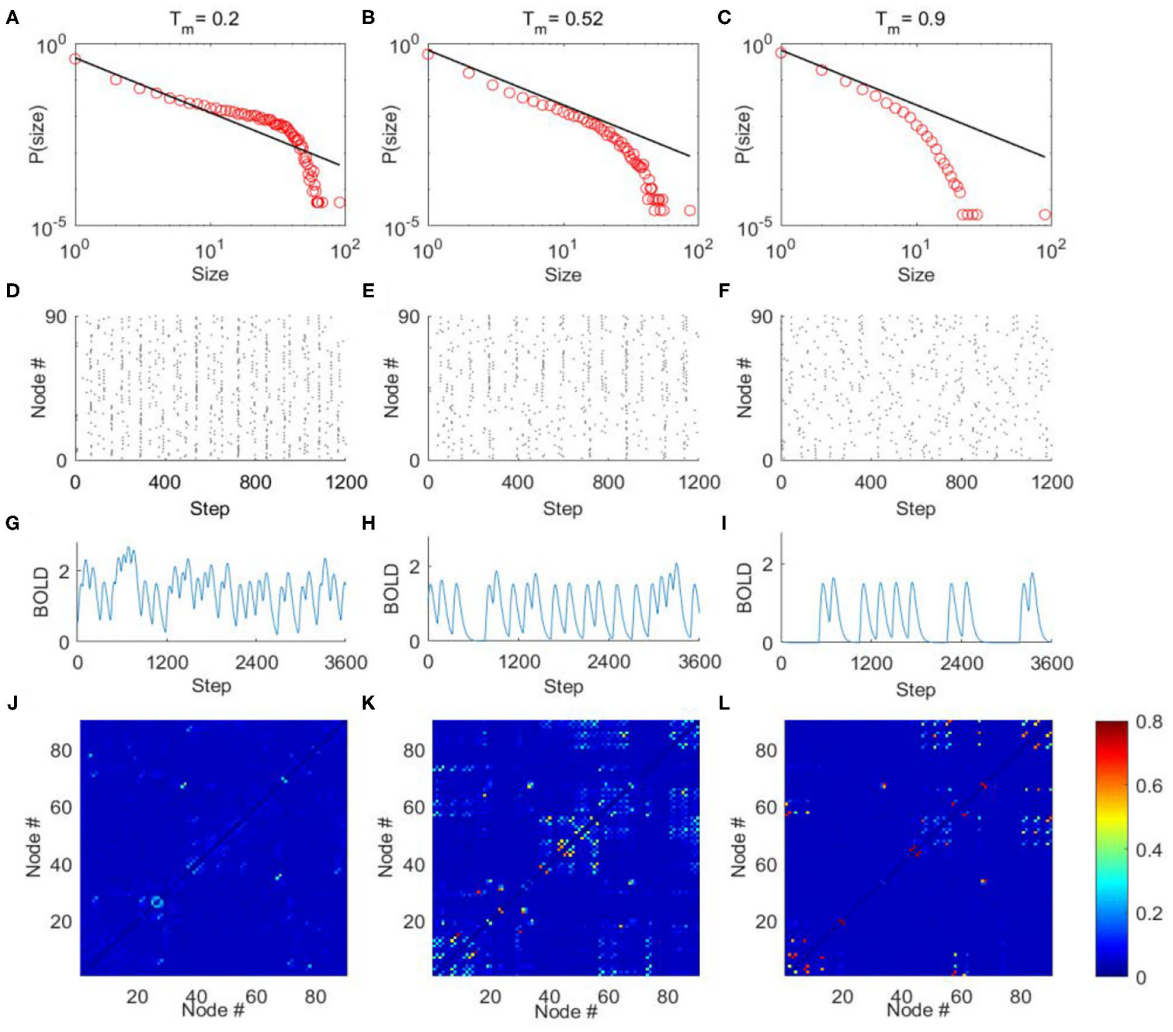
The avalanche activity in the DTI+GH brain network model, the simulated BOLD signals, and FC matrices. Through tuning the excitation threshold *T*_*m*_, the mode can exhibit typical avalanche distribution corresponding to supercritical **(A)**, critical **(B)**, and subcritical **(C)** dynamics. The horizontal axes are the size of avalanches, and vertical axes are the corresponding probability. The black lines in **(A**–**C)** indicate power law with exponent = −1.5. **(D**–**F)** The raster plots of spatial-temporal excitation distributions corresponding to **(A**–**C)**. The dots in the raster plots represent the excitation of the nodes (i.e., in state *E*). **(G**–**L)** The typical simulated BOLD signals of an arbitrarily chosen nodes and simulated FC matrices from the DTI+GH brain model in the supercritical **(G,J)**, critical **(H,K)**, and subcritical **(I,L)** regimes. Scale bar indicates the FC strength among the nodes in the model. The parameters of GH model in simulations are *r*_1_ = 0.005, *r*_2_ = 0.98, and *n*_*delay*_ = 55.

The criticality refers to a balanced state between ordered and disordered and is characterized by power law distribution of avalanche activity (i.e., the avalanche size distribution shows no characteristic scale). The supercritical state refers to the ordered states that are characterized by avalanche with large size, whereas the subcritical state refers to the disordered states that are characterized by avalanches with small size (Beggs and Plenz, 2003; Tagliazucchi et al., 2012; Shriki et al., 2013). In our model, we calculated the avalanche size distribution from the spatiotemporal patterns of excited nodes for different excitation threshold *T*_m_ (“**METHOD AND MATERIALS**,” “**Avalanche Detection**”). When *T*_m_ is low, the nodes in the model are excited easily, and their activities are highly synchronized to result in a rather ordered state (Figure 4D). Thus, the activities tend to form avalanches with large size to have a power-law slope with a heavier tail in the distribution (Figure 4A), indicating the supercritical dynamics. Whereas, when *T*_m_ is high, the nodes in the network are less excitable, and their activities are random and less synchronized (Figure 4F). Thus, the groups of activity are small and die out quickly, unlikely to form avalanches with large size, which is termed as subcritical regime (Figure 4C). In both cases, the size distribution of avalanches demonstrates a characteristic scale. However, with moderate *T*_m_ (*T*_*m*_ ≈ 0.52) the scale-free avalanche distribution emerges with an exponent of −1.5 (Figure 4B), and the system is perched between order and random (Figure 4E).

We then convolved the activities of each node in the model with the hemodynamic response function to generate 90 simulated BOLD time series (“**METHOD AND MATERIALS**,” “**DTI+GH Brain Network Model**”). Typical BOLD signals of arbitrarily chosen brain regions for supercritical, critical, and subcritical regimes are demonstrated in Figures 4G–I, respectively. The Hurst exponent calculated from these simulated BOLD signals yields its largest value at the critical point (Supplementary Figure 2A). The FC matrices corresponding to different regimes were then obtained by calculating the correlation coefficients among 90 simulated BOLD time series as before. We compared the similarity between simulated FC matrices and experimental FC networks and found the maximal similarity occurs around the critical point (Supplementary Figure 3A). It is also seen that when the system is poised at the critical point, the FC matrix exhibits patterns which is similar to the DTI structural connections (Figure 4K), whereas the supercritical and subcritical dynamics fail to replicate the DTI structural connections (Figures 4J,L). This phenomenon has been systematically studied with computer modeling and experiment on propofol-induced departure from critical dynamics (Tagliazucchi et al., 2016). It was argued that the functional organization of the brain is constrained and enabled by the unique structural organization (Tagliazucchi et al., 2016), and the spontaneous brain activity can be understood as an ever-transient exploration of the repertoire of paths offered by structural connections (Deco et al., 2014a; Tagliazucchi et al., 2016). The critical dynamics of the system would allow a more widespread exploration of all possibilities offered by the structural connections, makes FCs better reproduce its structural connections [see Ref. (Tagliazucchi et al., 2016) for a vivid explanation].

### Optimal Organization of the FC Network at Criticality

**Figure 5:**
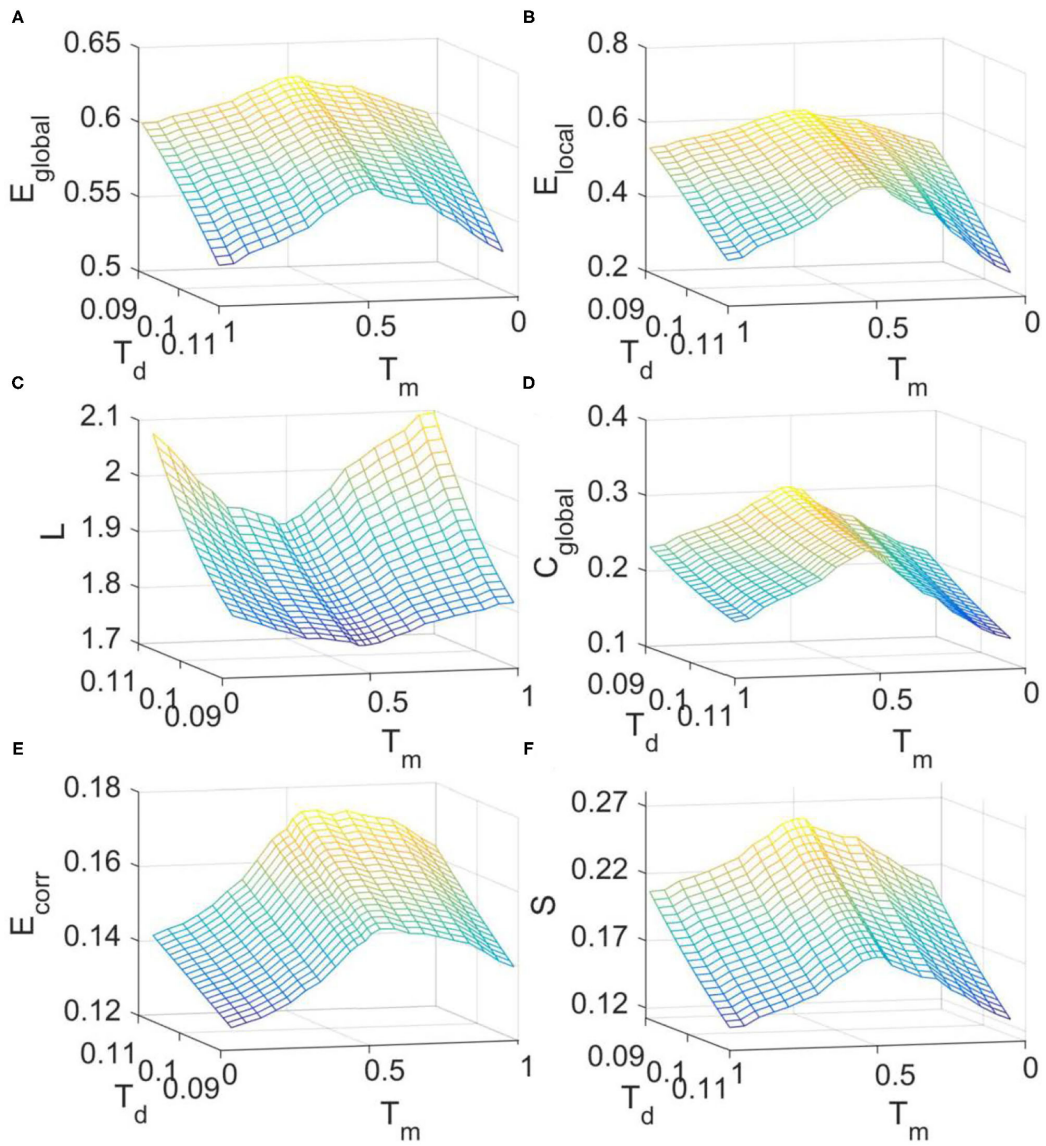
The dependence of FC network topological metrics simulated with DTI+GH model on the excitation threshold *T*_*m*_ and binarizing threshold *T*_*d*_. **(A)** Global efficiency. **(B)** Local efficiency. **(C)** Characteristic path length. **(D)** Clustering coefficient. **(E)** Mean connection strength. **(F)** Sparsity. The results were obtained by averaging results from 1,000 times of simulation. In each simulation, the obtained raw BOLD signals were sampled every 140 iteration steps to achieve the simulated BOLD time series of 200 time points.

We then investigated the changes of FC network metrics across the transition from supercritical to subcritical regime in the DTI+GH model. The simulated FC matrices were binarized with threshold *T*_d_ and the corresponding metrics were calculated in the small-world regime as before (Supplementary Figure 1B). It is seen from Figure 5 that around the critical point (*T*_m_ = 0.52), all the network metrics are maximized except for the characteristic path length which is minimized (Figure 5C). These results imply that critical dynamics can optimize brain FC network organization and the departure from criticality will cause the disruption of this optimal balance between integration and segregation.

It was also seen from Figure 5 that with the increase of binarizing threshold *T*_*d*_, global efficiency (Figure 5A), local efficiency (Figure 5B), clustering coefficient (Figure 5D), and sparsity (Figure 5F) decrease, whereas the characteristic path length (Figure 5C) and the mean connection strength (Figure 5E) increase. The dependence of these network metrics on the binarizing threshold *T*_*d*_ predicted by our model is in line with that obtained from the fMRI data (Figure 2), and that reported by other researchers (Liu et al., 2008).

### Local *E*/*I* Ratio Tunes Critical Dynamics in the DTI+EI Network Model

**Figure 6:**
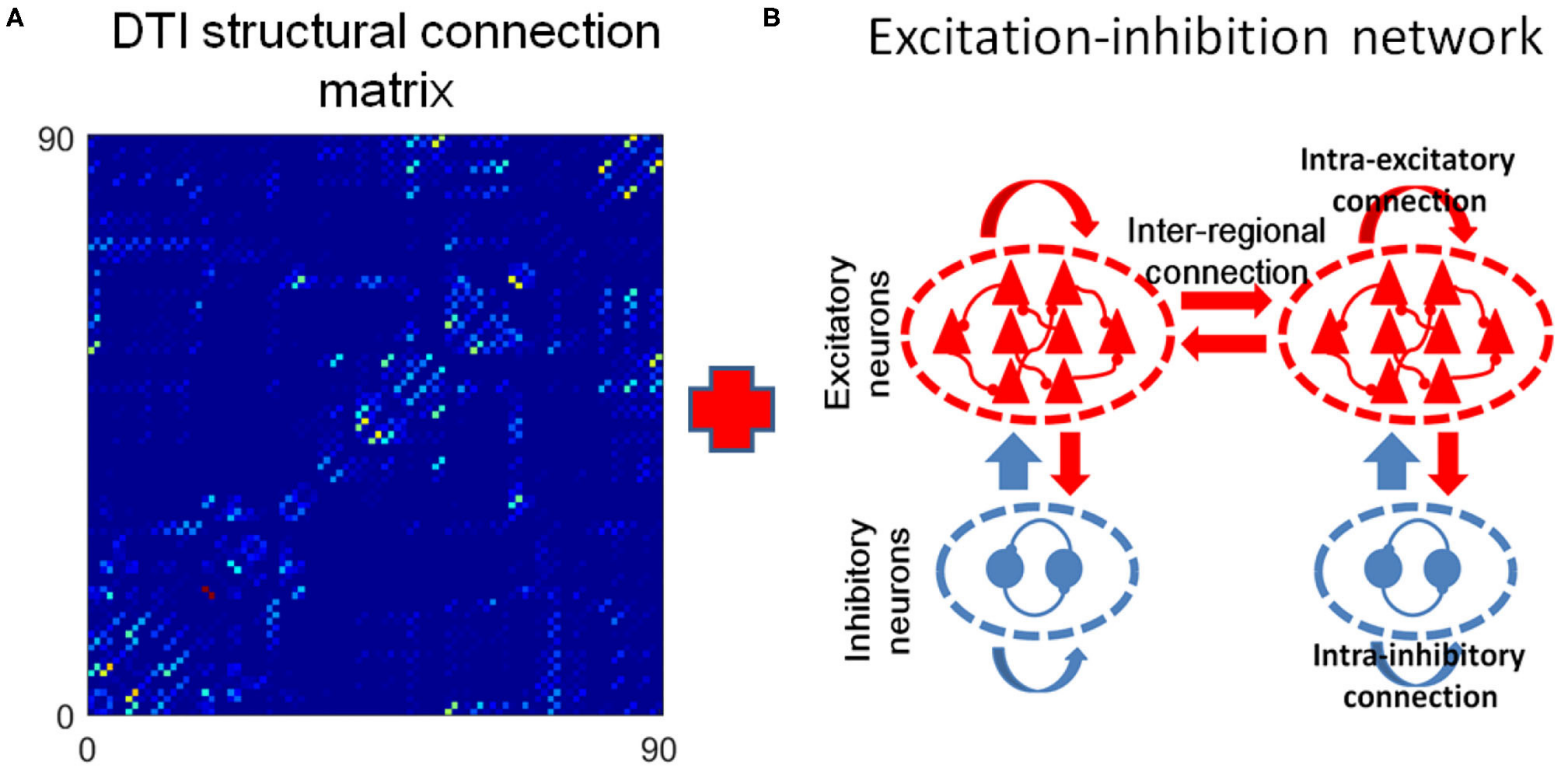
The DTI+EI whole brain model. **(A)** The DTI structural connection matrix. **(B)** Example of two excitation-inhibition (EI) neuronal networks that represent two brain regions. Each regional neuron network consists of one excitation neuron pool and one inhibitory neuron pool. They are 80% excitatory neurons and 20% inhibitory neurons in the network. The excitatory neurons send our only excitatory synapses to other neurons and the inhibitory neurons send out only inhibitory synapses. The two EI networks are coupled only by excitatory inter-regional connections.

Next, we built a large-scale brain functional model based on DTI structural connection data and EI neuronal networks (Figure 6). In this model, the neural activity in each region is modeled with a neuronal network composing 100 excitatory (*E*) and 25 inhibitory (*I*) neurons so that the ratio of number of excitatory neurons to that of inhibitory ones is 4:1. The single neuron dynamics is modeled with Izhikevich cortical spiking neuron model, which is computationally efficient and biologically plausible (Izhikevich, 2004). The neurons in each EI networks are connected with a probability of 0.5. The excitatory neurons send out only excitatory synaptic connections to other neurons and inhibitory neurons send out only inhibitory ones. In the simulation, we systematically change the *E*–*E* connection strength but fixed other ones and define the ratio of *E*–*E* to *I*–*I* synaptic connection strength as the local *E*/*I* ratio. The number of excitatory neurons that establish inter-regional connections is proportional to the number of fibers connecting corresponding brain regions (see “**METHOD AND MATERIALS**,” “DTI+EI Whole Brain Model” for details).

**Figure 7:**
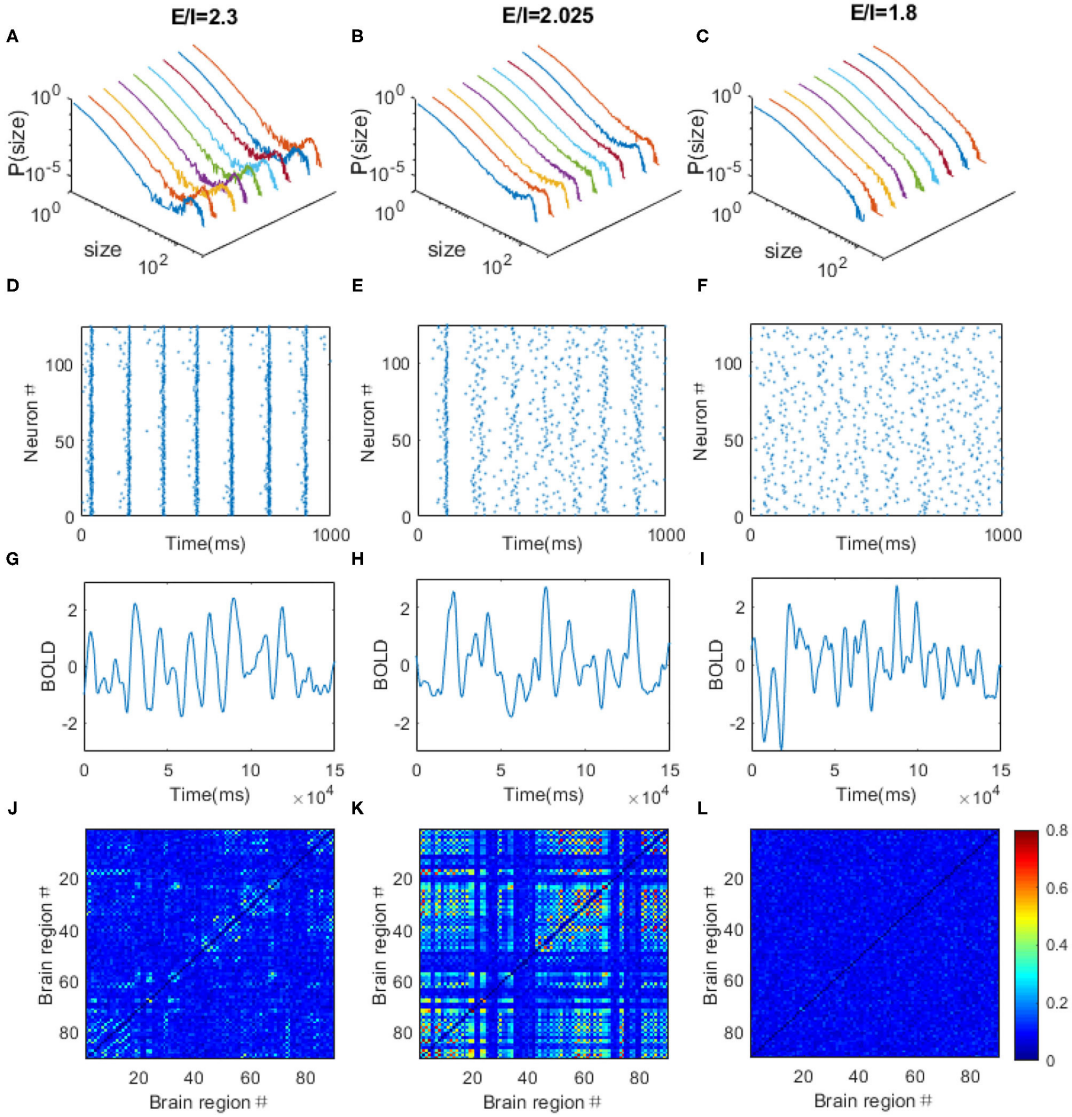
The dynamical behaviors of the DTI+EI brain model. Through tuning the *E*/*I* ratio, the model can exhibits supercritical **(A)**, critical **(B)**, and subcritical **(C)** avalanche dynamics in each regions. Colored lines are corresponding to different brain regions chosen arbitrarily. **(D**–**F)** The raster plots of spatial-temporal firing distributions of an arbitrarily chosen brain regions corresponding to **(A**–**C)**. The firings are more synchronized in the supercritical regions **(D)**, but the firings are rather random when the system is subcritical with decreased excitatory connections **(F)**. The critical dynamics is characterized with moderate synchrony **(E)**. The dots in the raster plots indicate the firing of the neurons. **(G**–**L)** The typical simulated BOLD signals of an arbitrarily chosen brain region and simulated FC matrices from the DTI+EI brain model in the supercritical **(G,J)**, critical **(H,K)** and subcritical **(I,L)** regimes. Scale bar indicates the FC strength among the nodes in the model.

Through adjusting the local *E*/*I* ratio in each region simultaneously, we observed the power law distribution of avalanche activities with exponent of −1.5 within each brain region when the *E*/*I* ratio is around 2.025 (Figure 7B), indicating the critical dynamics of the system. Whereas, the system is supercritical when the *E*/*I* ratio is high (Figure 7A) but subcritical when the *E*/*I* ratio is low (Figure 7C). The spikes of the neurons are quite synchronized when the system is supercritical, especially for the excitatory neurons because of the strong excitatory connections among them (Figure 7D), but the firings are rather random when the system is subcritical with decreased excitatory connections (Figure 7F). The critical dynamics of the system exhibits moderate synchrony where synchronous firing occurs occasionally among excitatory neurons (Figure 7E). After, taking spike rate in each region as the input, we used the Balloon-Windkessel hemodynamic model to generate simulated BOLD signal for each region (see “**METHOD AND MATERIALS**” for details). Figures 7G–I demonstrates the examples of simulated BOLD signals from arbitrarily chosen brain regions for each case. Again, the Hurst exponent of these simulated BOLD signals exhibits its maximal values at the critical point (Supplementary Figure 2B). We then obtained the 90 × 90 FC matrices from the 90 simulated BOLD time series for each regime. We observed FC patterns emerge when the system is critical (Figure 7K). However, the pattern vanishes if the system is poised in the supercritical (Figure 7J) or subcritical regimes (Figure 7L). Again, the simulated FC matrices are most close to experimental FC network when the model is at its critical point (Supplementary Figure 3B).

### Dependence of FC Network Metrics on Local *E*/*I* Ratio in the DTI+EI Model

**Figure 8:**
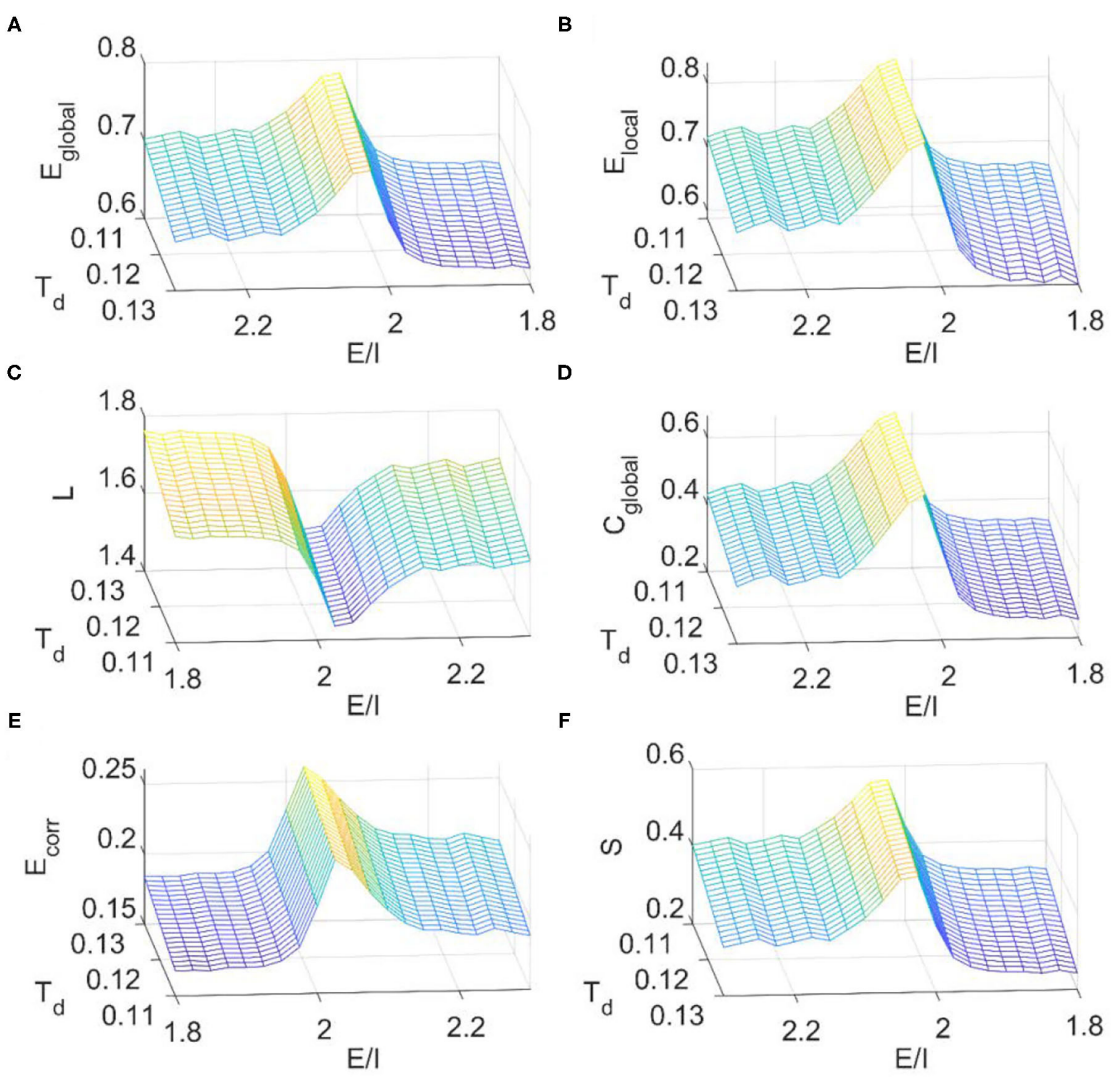
The dependence of topological metrics of the FC network on the thresholding value *T*_*d*_ and *E*/*I* ratio in the DTI+EI whole brain model. **(A)** Global efficiency. **(B)** Local efficiency. **(C)** Characteristic path length. **(D)** Clustering coefficient. **(E)** Mean connection strength. **(F)** Sparsity. The results were obtained by averaging results from 10 times of simulation. Each simulation last for 480 s with a time step of 1 ms and the first 180 s was removed for stability. The obtained raw BOLD signals were then normalized and sampled at a rate of 0.5 Hz.

We then calculated the dependence of simulated FC network metrics on the *E*/*I* ratio and the binarizing threshold *T*_*d*_. The range of *T*_*d*_ for small-world regime was determined in the same way as before (Supplementary Figure 1C). It is seen from Figure 8 that the *E*/*I* ratio, at where the critical dynamics emerges, maximizes the global efficiency (Figure 8A), local efficiency (Figure 8B), clustering coefficient (Figure 8D), mean connection strength (Figure 8E), and sparsity (Figure 8F) but minimizes the characteristic path length (Figure 8C). These results further suggest that the local *E*/*I* ratio could adjust the global brain dynamics to the critical state, so as to achieve the balance between segregation and integration in FC networks. On the other hand, this optimal organization of FC networks could be damaged when the optimal *E*/*I* ratio is altered. As the binarizing threshold *T*_*d*_ increases, global efficiency, local efficiency, clustering coefficient, and sparsity decrease, but the characteristic path length and the mean connection strength increase. The dependence of these metrics on the binarizing threshold is in line with our above results from fMRI data analysis (Figure 2), the DTI+GH brain model (Figure 5) and that reported by other researchers (Liu et al., 2008).

## Discussion and Conclusion

It has been shown that a network with shorter characteristic path length benefit the global efficiency, while a network with densely local connectivity benefit the local efficiency. Only in the small-world region, i.e., low characteristic path length combined with large clustering coefficient, does the network display globally and locally efficient at the same time (Latora and Marchiori, 2001). Recent analysis of human brain functional networks derived from EEG/MEG and fMRI experiments showed that these networks exhibit prominent small-world organization. Through forming intrinsically densely connected and strongly coupled local network communities, the small-world topology facilitates functional segregation. Meanwhile, by enabling global communication between communities through network hubs, it also promotes functional integration (Bassett and Bullmore, 2006; Sporns, 2013). In this work, through resting-state fMRI data analysis and computational model of whole brain dynamics, we demonstrated that the critical dynamics favors this optimal organization of FC networks, and failure of critical dynamics causes the collapse of balance between segregation and integration in the network by increasing the characteristic path length and decreasing the cluster coefficient.

Critical dynamics in brains has been observed at brains at different levels, from single neuron to the whole brain levels, with different recoding techniques (Shew et al., 2009; Gal and Marom, 2013; Gollo et al., 2013; Mora et al., 2015). Recent work with resting-state fMRI data analysis demonstrated the existence of large-scale critical dynamics, hallmarked by scale-free avalanche activity, in the human cortex (Tagliazucchi et al., 2012). Beside these observations, the critical brain hypothesis argued that criticality benefits neural information processing in many ways, e.g., the maximal information transmission and storage capabilities (Shew et al., 2011; Timme et al., 2016). However, these arguments usually defined the advantages of criticality in the general framework of information-theoretic [e.g., mutual information entropy (Shew et al., 2011)], neural dynamics [e.g., maximal dynamic range (Shew et al., 2009; Gautam et al., 2015), or the number of the metastable states in the energy landscape (Shew et al., 2009)], but their direct relations to brain functions are unclear. The FC network metrics have been related to many factors that affect brain functional performance, e.g., intellectual performance (van den Heuvel et al., 2009), aging (Wang et al., 2010), and a variety of brain diseases (Stam et al., 2007; Liu et al., 2008; Wang et al., 2009; Sanz-Arigita et al., 2010; Zhang et al., 2011; Rudie et al., 2013). Furthermore, it is believed that both segregated and integrated information processing are facilitated by the small-world topology of FC networks. The information transmission efficiency is maximized with this small-world topology, with their high clustering coefficient for segregated processing and short characteristic path length for integrated processing. Meanwhile, the disrupted network organization was found in neuropsychiatric disorders, usually characterized by increased characteristic path length and decreased cluster coefficient, and these changes were correlated with symptom severity in clinical-scale examinations (Stam et al., 2007; Liu et al., 2008; Wang et al., 2009; Zhang et al., 2011; Rudie et al., 2013). In our work, we found the critical dynamics maximizes clustering coefficient but minimizes the characteristic path length and yields both maximal local and global efficiency of the FC network. So our findings presented in this work not only uncovered the possible underlying dynamics from which the small-world FC network organization emerges but also revealed the advantage of large-scale critical dynamic in information processing at the whole brain level.

It is well-established that the EI balanced is critical for the forming of critical dynamics in healthy brains (Poil et al., 2012; Yang et al., 2012), and the neural systems may achieved this balanced state through synaptic plasticity (de Arcangelis et al., 2006; Stepp et al., 2015). On the contrary, the EI imbalance hypothesis has been postulated to underlie brain dysfunction across neurodevelopmental and neuropsychiatric disorders (Canitano and Pallagrosi, 2017; Foss-Feig et al., 2017). It was recently demonstrated that regulating the local *E*/*I* ratio crucially changes not only the characteristics of the emergent resting activity but also evoked activity. It also gives a more robust prediction of resting state FCs. Furthermore, it enhances the information capacity and the discrimination accuracy in the global networks (Deco et al., 2014b). These arguments have led to another hypothesis that criticality is a signature of healthy neural systems (Massobrio et al., 2015). In this study, we demonstrated that through tuning the *E*/*I* ratio of the brain model, the system could be poised at the critical point, and at this critical point, the functional integration and segregation of brain FC network is optimized. Considering the well-reported disruption of FC network in brain diseases, our modeling work with EI networks not only revealed the crucial role of the local *E*/*I* ratio in the forming of the optimal organization of whole brain FC networks but also provided supportive evidence for the hypothesis of EI imbalance by linking it with disruption of FC organization at the whole brains level, which has been observed in many brain diseases.

One attractive point and also the limitation of EI imbalance hypothesis is that brain disorders can be arranged in an imaginary line around the optimal point that balances excitation and inhibition. The limitation for unidimensionality of the EI imbalance has been discussed recently and it was argued that the higher dimensional models can better capture the multidimensional computational functions of neural circuits (O'Donnell et al., 2017). Therefore, EI balance may be not the only factor that is responsible for aberrant neural activity and FC network organization in diseased brains. Our results from DTI+GH model suggested that the general conclusion in this work still holds even in this case, since optimal organization of FC networks can emerge from critical dynamics without EI connections. These results implies the possibility of utilizing criticality to bridge the gap between altered FC organization caused by diseases at the whole brain level and aberrant neural activity described by higher dimensional models at the circuit level, rather than one-dimensional EI model.

However, there are several limitations in the current study. In the fMRI data analysis, the Hurst exponent was used as an indicator of criticality. However, it cannot distinguish the super- or subcritical state of the system. The full solution for this problem requires the calculation of avalanche size distribution, branching ratio, as well as mean synchronization, as had done in EEG (Meisel et al., 2013). However, though the scale-free distribution of avalanche has been observed with fMRI (Tagliazucchi et al., 2012), the applicability of this method alone to identify super- or subcritical dynamics is still questionable. The major concern is that unlike EEG, fMRI does not measure neural activity directly but *via* the changes of BOLD signals. Therefore, future investigations that combines EEG and fMRI are necessary to validate the conclusions drawn from this study (Fagerholm et al., 2015).

It is also noticed that after the critical dynamics in our models is established, there is quite a few parameters that must be determined to obtain the simulated BOLD signals. Due to the simplifications made in the models, the neural activities produced by models are not exactly in the same time scale as in the real brains. Therefore, though we used the standard parameters for hemodynamic response function in models [it is also noticed that though these function and model were used widely in simulation of BOLD signals (Deco et al., 2011; Haimovici et al., 2013; Tagliazucchi et al., 2016), they were actually proposed for task-related hemodynamic response, not for resting state], simulation parameters (such as fMRI sampling rate, duration for scanning session) are not exactly the same as these in the experiment. Therefore, our results in this work requires further test with more detailed simulations of whole brain neural dynamics, as well as more detailed simulation of hemodynamic response in the resting state fMRI (Rangaprakash et al., 2017).

In this study, we tested the hypothesis that critical dynamics is responsible for optimal organization of brain FC networks which is usually featured with “small worldness.” We found that the LRTCs of the BOLD signals measured with Hurst exponent is significantly correlated with the topological metrics of the FC networks, suggesting there exists an optimal dynamics for the brain FC network organization. Based on the inter-regional structural connection provided by DTI data, we built two kinds of whole brain dynamics model, using either simple cellular automaton, or more biological plausible neuronal networks with EI synaptic connections. In these models, we demonstrated that the critical dynamics could optimize the brain FC network organization through maximizing its cluster coefficient, while minimizing the shortest characteristic path length, so to achieve highest efficiency information transmission in the brain. We further showed that the local *E*/*I* ratio would have a great impact on critical dynamics and the organization of whole brain FC networks, suggesting imbalanced EI in brain circuitry may be responsible for the loss of small worldness in FC networks of brain disorder.

In conclusion, we demonstrated that the critical dynamics could optimize the brain FC network organization through maximizing its cluster coefficient, while minimizing the characteristic path length, so to achieve highest efficiency information transmission in the brain. Furthermore, imbalanced EI in brain circuitry may be responsible for the loss of the optimal organization in FC networks observed in brain disorder. Our findings revealed the crucial role of large scale critical dynamics in the forming of optimal FC network organization for efficient information processing, and potential relationship between local EI imbalance and the disrupted small-world organization. We hope that in the future these findings could not only lead to fundamental understanding on human brain function in health and its alterations in disease, but also help to develop whole brain computer models that could account for these alterations in brain disorder.

## Methods and Materials

### fMRI Data Acquisition and Preprocessing

One hundred right-handed healthy subjects (mean age: 31.2 ± 8.8 years, range: 15–70 years, 63 males) participated in the study. The degree of education is from 0 to 23 years (mean: 8.5 years). All participants were screened to ensure they were free of neurological or psychiatric disorders. The data was acquired using a Siemens Trio 3.0 Tesla MRI scanner at the Second Hospital of Lanzhou University. All subjects provided written informed consent prior to the study which was approved by the medical ethics committee of the Second Hospital of Lanzhou University in accordance with the 1964 Declaration of Helsinki and its later amendments or comparable ethical standards. Participants were instructed to relax and keep their eyes closed, remain as motionless as possible, and not to think of anything in particular. Both functional and high-solution structural MRI were applied to all participants. T2^*^-weighted resting-state fMRI data were acquired using a gradient-echo EPI sequence, TR = 2 s, TE = 30 ms, slice thickness = 3 mm, gap = 0.99 mm, FOV = 240 mm, matrix size = 64 × 64. The scans lasted 360 s (180 volumes). High-resolution T1-weighted images were acquired with a magnetization prepared rapid gradient echo sequence, TR = 2 s, TE = 2.67 ms, inversion time = 900 ms, slice thickness = 1 mm, gap = 1 mm, FOV = 220 × 220 mm, matrix size = 256 × 224.

Preprocessing of fMRI data was performed using Statistical Parametric Mapping (SPM) 8 (http://www.fil.ion.ucl.ac.uk/spm) and the Data Processing Assistant for Resting-State fMRI (DPARSF) within the Data Processing and Analysis for Brain Imaging (DPABI) (Yan and Zang, 2010). Volumes were corrected for slice timing and head movements, and five subjects were excluded for excessive head movement (>3 mm or >3°) during the scan. After spatial normalization (Montreal Neurological Institute space), resampling (3 mm isotropic voxels), and spatial smoothing (4 mm, full-width, half-maximum Gaussian kernel), volumes were preprocessed using linear trend subtraction and temporal filtering (0.01–0.08 Hz). In addition, using the general linear regression, nuisance regressors including head motions, global mean signals, white matter signals, and cerebrospinal fluid signals were regressed out from the fMRI time series.

### The DTI Data Acquisition and Processing

In this study, the DTI data was obtained from IMAGEN consortium, which included 142 healthy participants (76 females, age: 14.5 ± 0.2 years). The detailed information of data acquisition could be found in Ref. (Schumann et al., 2010). The DTI data were corrected for motion and eddy current distortion using FMRIB Software Library v5.0 (FSL, http://www.fmrib.ox.ac.uk/fsl) (Jenkinson et al., 2012). In addition, we extracted the brain mask from the B0 image. We used the TrackVis (Wang et al., 2007) to perform the fiber tractography with the deterministic tracking method. Maps of fractional anisotropy (FA) were computed from the DTI data. The regions of interest (ROIs) were determined by the AAL atlas-based T1 image from each subject (Tzourio-Mazoyer et al., 2002), using the PANDA suite (Cui et al., 2013). Finally, between each pair of ROIs, we assessed the fiber number to construct the DTI structural connection matrix.

### Hurst Exponent

We use the Hurst exponent to measure the extent of long-range memory of the BOLD time series, either from the fMRI data or from the simulation with both brain models. The Hurst exponent is estimated using the method of classical rescaled range (RS) method (Blythe and Nikulin, 2017):

1. Divide the time series ${\left\{y\left(t\right)\right\}}_{t=1}^{T}$ into *M* subseries by choosing an appropriate number *n*, and each subseries has a window length of *n*.
2. For each subseries (*m* = 1, 2, *M*), calculate the local statistic $LS_{n,m}=\frac{R_{n,m}}{S_{n,m}}$. The range of *m*th subseries *R*_*n, m*_ = *max*(*Z*_1_, *Z*_2_, …, *Z*_*n*_) − min(*Z*_1_, *Z*_2_, …, *Z*_*n*_), where $Z_{k}=\sum_{t=1}^{k}(y_{t,m}-y_{n,m})$. *S*_*n, m*_ is the standard deviation of *m*th subseries, which is calculated as $S_{n,m}=\sqrt{\frac{1}{n}\sum_{t=1}^{n}(y_{t,m}-y_{n,m}{)}^{2}}$. Then by averaging over all subseries, we obtain the global statistic, i.e., $SS_{n}=\frac{1}{M}\overset{M}{\underset{m=\;1}{\sum }}LS_{n,m}$.
3. Through changing *n* and repeating the previous steps, we obtain a series of *SS*_*n*_ corresponding to a different choice of *n*.
4. The Hurst exponent is estimated by fitting the power law $SS_{n}\approx Cn^{H}$ to the data. This can be done by running a double logarithm regression for a series of *SS*_*n*_ corresponding to different values of *n*.

In the calculation, the global Hurst exponent of the whole brain level was obtained by average the local Hurst exponent across 90 brain regions in both fMRI data analysis and the DTI+EI model. Whereas, in the DTI+GH models, to obtain the stable estimation of Hurst exponent of the systems, we first averaged the 90 simulated BOLD time series and then calculated its Hurst exponent.

### Network Metrics

First, we used the AAL template to extract from 90 brain regions 90 time series, each of which is the averaged BOLD signals across all the voxels in each region. The correlation coefficients for each pair of the time series was then calculated to build the FC matrix z(*i, j*) (*i, j* = 1, 2, 90), in which each off-diagonal element is the correlation coefficient between a pair of brain regions. The FC network was constructed by setting a threshold *T*_*d*_ to each element in the absolute FC matrix:

(1) $$\begin{array}{l} a_{ij}=\{\begin{array}{ll} 1 & \text{if }|z\left(i,j\right)|\geq T_{\text{d}}\\ 0 & \text{otherwise} \end{array} \end{array}$$

The network metrics of a FC network with *n* nodes was calculated as follows:

The degree of a node *i* is defined as the number of its direct neighbors:

(2) $$\begin{array}{l} k_{i}=\sum_{j\in N}a_{ij} \end{array}$$

where *a*_*ij*_ is the connection between nodes *i* and *j*, *a*_*ij*_ = 1 when they are directly linked, *a*_*ij*_ = 0 if not.

The connectivity strength of the node *i* is:

(3) $$\begin{array}{l} E_{i\text{\_}\text{corr}}=\frac{1}{k_{i}}\underset{j\in N}{\sum }|z\left(i,j\right)|\cdot a_{ij}, \end{array}$$

which is a measure to evaluate the strength of the connectivity between node *i* and the nodes connected to it. The connectivity strength of a network is:

(4) $$\begin{array}{l} E_{\text{corr}}=\frac{1}{n}\underset{i\in N}{\sum }E_{i\text{\_}\text{corr}}. \end{array}$$

The ratio of the number of existing edges to the number of maximum possible number:

(5) $$\begin{array}{l} S=\frac{1}{n\left(n-1\right)}\underset{i\in N}{\sum }k_{i}, \end{array}$$

is defined as the sparsity of the network.

Characteristic path length measures the extent of average connectivity or overall routing efficiency of the network (Sanz-Arigita et al., 2010), which is defined as

(6) $$\begin{array}{l} L=\frac{1}{n}\underset{i\in N}{\sum }L_{i}=\frac{1}{n}\underset{i\in N}{\sum }\frac{\sum_{j\in N,j\neq i}d_{ij}}{n-1}, \end{array}$$

in which $d_{ij}=\underset{a_{uv}\in g_{i↔j}\;}{\sum }a_{uv}$ is the shortest path length between nodes *i* and *j* with the shortest way *g*_*i*↔*j*_ and *L*_*i*_ is the mean shortest path length of node *i*.

Global efficiency is a measure of the efficiency of parallel information transfer in the network at the global level:

(7) $$\begin{array}{l} E_{global}=\frac{1}{n}\underset{i\in N}{\sum }E_{i}=\frac{1}{n}\underset{i\in N}{\sum }\frac{\sum_{j\in N,j\neq i}{d_{ij}}^{-1}}{n-1}. \end{array}$$

Local efficiency, which measures the efficiency at the local level, is defined as:

(8) $$\begin{array}{l} E_{local}=\frac{1}{n}\underset{i\in N}{\sum }E_{loc,i}=\frac{1}{n}\underset{i\in N}{\sum }\frac{\sum_{j,h\in N,j\neq i}a_{ij}a_{ih}{\left[d_{jh}\left(N_{i}\right)\right]}^{-1}}{k_{i}\left(k_{i}-1\right)}, \end{array}$$

where *d*_*jh*_(*N*_*i*_) is the shortest path length between nodes *j* and *h* which should be the nodes directly connected to node *i*.

Clustering coefficient measures the possibility that any two neighbors of one node are also connected, i.e., the extent of the local density of the network:

(9) $$\begin{array}{l} C_{global}=\frac{1}{n}\underset{i\in N}{\sum }C_{i}=\frac{1}{n}\underset{i\in N}{\sum }\frac{2t_{i}}{k_{i}\left(k_{i}-1\right)}, \end{array}$$

where $t_{i}=\frac{1}{2}\underset{j,h\in N}{\sum }a_{ij}a_{ih}a_{jh}$ is the number of triangles around node *i*.

The network metrics were calculated in a range of threshold that is small enough to assure the mean number of connected nodes within each group was > 89, yet large enough so that the small worldness of network still holds, i.e., the global efficiency of the normal networks is less than the global efficiency of the random networks.

### DTI+GH Brain Network Model

The DTI+GH brain network model used in this study was adapted from the model proposed by Haimovici et al. (2013). The coupling strength *w*_*ij*_ between any two nodes *i* and *j* was given by the corresponding element in the DTI structural connection matrix multiplied by 0.01. Each node was modeled with the Greenberg-Hastings (GH) dynamics. The detailed description of GH dynamics could be found in Ref. (Haimovici et al., 2013). We binarized the time series of each node by assigning state *E* = 1 and the rest of the states into 0 s. To model the brain neurometabolic coupling, we then convolved the binarized time series with a hemodynamic response function (Henson and Friston, 2007):

(10) $$\begin{array}{l} f\left(t\right)={\left(\frac{t-o}{d}\right)}^{p-1}{\left(\frac{\exp\left(-\left(t-o\right)/d\right)}{d\left(p-1\right)!}\right)}^{p-1}, \end{array}$$

where *d* = 0.6 is the time-scaling, *o* = 0 is the onset delay, and *p* = 3 is an integer phase-delay (the peak delay is given by *pd*, and the dispersion by *pd*^2^). The obtained raw BOLD signals were sampled every 140 iteration steps to have the simulated BOLD time series of 200 time points.

### DTI+EI Whole Brain Model

In this model, each brain region is modeled by an EI neuronal network comprising 100 excitatory and 25 inhibitory neurons. As in the mammalian neocortex, the ratio of excitatory to inhibitory cells is 4 to 1 (DeFelipe et al., 2002). The connection probability between these neurons is set to 0.5. Then we use the Izhikevich model to produce single neuron dynamics (Izhikevich, 2004):

(11) $$\begin{array}{l} \frac{{\text{dv}}^{\text{i}}}{\text{dt}}=0.04{\left({\text{v}}^{\text{i}}\right)}^{2}+5{\text{v}}^{\text{i}}+140-{\text{u}}^{\text{i}}+{\text{I}}_{\text{synapse}}+\xi (\text{t}), \end{array}$$

(12) $$\begin{array}{l} \frac{{\text{du}}^{\text{i}}}{\text{dt}}=a\left({\text{bv}}^{\text{i}}-{\text{u}}^{\text{i}}\right), \end{array}$$

(13) $$\begin{array}{l} {\text{If v}}^{\text{i}}\geq 30\text{ mV, then}\{\begin{array}{c} v^{i}\leftarrow c\\ u^{i}\leftarrow u^{i}+d \end{array} \end{array}$$

where *v*^*i*^ and *u*^*i*^ represent the *i*th neuron's membrane potential and recovery, respectively. The parameters *a, b, c*, and *d* are set to model either excitatory (0.02, 0.2, −65 + 15*r*^2^, 2.8−6*r*^2^) or inhibitory (0.02 + 0.08*r*, 0.2–0.05*r*, −65, 2) neurons. To introduce some variability in the neuronal population, the variable *r* is drawn from a uniform distribution *U*(0,1). *I*_*synapse*_ represents synaptic currents this neuron receives from other neurons. ξ(*t*) is the background Gaussian white noise with 〈ξ(*t*)〉 = 0 and 〈ξ(*t*)ξ(*t*′)〉 = Dδ(t-t'), where the noise intensity *D* = 25 for excitatory neurons and *D* = 6.25 for inhibitory neurons. Equation (13) models the after-spike reset behavior when the membrane potential *v*^*i*^ exceeds a threshold. This model is widely used in large-scale neuronal network modeling because of its computational efficiency and biological plausibility (Izhikevich, 2004).

In our model, the synaptic current received by one neuron (*I*_*synapse*_) can be divided into two parts: $I_{intra<uscore>synapse}^{i}$ is the synaptic current the *i*th neuron receives from other neurons in this brain region, which is written as:

(14) $$\begin{array}{l} {\text{I}}_{\text{intra}<\text{uscore}>\text{synapse}}^{\text{i}}\text{=}\sum_{\text{j}\neq \text{i}}{\text{g}}_{\text{E}\to \text{E},\text{E}\to \text{I},\text{I}\to \text{E},\text{I}\to \text{I}}^{\text{ij}}\;\delta \left(\text{t}-{\text{t}}_{\text{spike}}^{\text{j}}\right), \end{array}$$

where $t_{spike}^{j}$ is the time instant when the presynaptic neuron *j* that exerts synaptic connection to the neuron *i* fires a spike. The summation runs across all the neurons that exert synaptic connection to the neuron *i*. The intra-regional synaptic connecting strength is set as follow: $g_{E\to I}^{ij}=1$ if the *j*th neuron is excitatory and *i*th neuron is inhibitory; $g_{I\to E,I\to I}^{ij}=-1$ if the *j*th neuron is inhibitory no matter if the *i*th neuron is excitatory or inhibitory. In the simulation, we systematically varied the connections among excitatory neurons $g_{E\to E}^{ij}$ to change the local *E*/*I* ratio, which was defined as the ratio of $g_{E\to E}^{ij}$ to $g_{I\to I}^{ij}$.

The inter-regional connections are set only for excitatory neurons among the different brain regions. Therefore, $I_{inter<uscore>synapse}^{ij}=0$ if neurons *i* and *j* belong to different regions and at least one of them is an inhibitory neuron. The inter-regional connection probability of excitatory neurons in each pair of brain regions is proportional to their corresponding DTI structural connection strength and the maximum is set to be 0.5. Specifically, if the DTI connection between region *m* and *n* is *q*, then the excitatory neurons in these two brain regions have an inter-regional connection probability of 0.5*q*_mn_/*q*_max_, where *q*_max_ is the highest value in the DTI matrix. For example, for neuron *i* in one region, it receives inter-regional synaptic currents from neuron *j* in another region is written in the following form:

(15) $$\begin{array}{l} {\text{I}}_{\text{inter}<\text{uscore}>\text{synapse}}^{\text{ij}}\text{=}\sum_{\begin{array}{l} i\in M\\ j\in N \end{array}}{\text{g}}_{\text{E}↔\text{E}}^{\text{ij}}\;\delta \left(\text{t}-{\text{t}}_{\text{spike}}^{\text{j}}\right), \end{array}$$

where *E*↔*E* represents the inter-regional excitatory synaptic coupling. The inter-regional synaptic connection $g_{E↔E}^{ij}=\;0.15$ in the simulation.

For DTI+EI model, the fMRI BOLD signals are computed with Balloon-Windkessel hemodynamic model (Friston et al., 2003). The regional BOLD signal is driven by the collective neuronal activity of both excitatory and inhibitory neurons. For region *i*, we define neuronal activity *z*_*i*_ as the ratio of number of spikes to the number of neurons in the region within a time window of 1 ms. We assume *z*_*i*_ causes an increase in a vasodilatory signal *s*_*i*_ that increases the flow *f*_*i*_. The inflow *f*_*i*_ then causes changes in blood volume *v*_*i*_ and deoxyhemoglobin content *q*_*i*_:

(16) $$\begin{array}{l} \frac{{\text{ds}}_{\text{i}}(\text{t})}{\text{dt}}=\epsilon_{\text{i}}{\text{z}}_{\text{i}}-{\text{k}}_{\text{i}}{\text{s}}_{\text{i}}-\gamma_{\text{i}}\left({\text{f}}_{\text{i}}-1\right), \end{array}$$

(17) $$\begin{array}{l} \frac{{\text{df}}_{\text{i}}(\text{t})}{\text{dt}}={\text{s}}_{\text{i}}, \end{array}$$

(18) $$\begin{array}{l} \tau_{\text{i}}\frac{\text{d}{\text{v}}_{\text{i}}\left(\text{t}\right)}{\text{dt}}={\text{f}}_{\text{i}}-{\text{v}}_{\text{i}}^{1/\alpha }, \end{array}$$

(19) $$\begin{array}{l} \tau_{\text{i}}\frac{\text{d}{\text{q}}_{\text{i}}\left(\text{t}\right)}{\text{dt}}=\frac{{\text{f}}_{\text{i}}\left(1-{\left(1-\rho_{\text{i}}\right)}^{{\text{f}}_{\text{i}}}\right)}{\rho_{\text{i}}}-\frac{{\text{q}}_{\text{i}}{\text{v}}_{\text{i}}^{1/\alpha }}{{\text{v}}_{\text{i}}}, \end{array}$$

where ρ is the resting oxygen extraction fraction. Taken as a static non-linear function of volume and deoxyhemoglobin that comprises a volume-weighted sum of extra- and intravascular signals, the BOLD signal is then calculated as:

(20) $$\begin{array}{l} {\text{y}}_{\text{i}}={\text{V}}_{0}\left(7\rho_{\text{i}}\left(1-{\text{q}}_{\text{i}}\right)+2\left(1-\frac{{\text{q}}_{\text{i}}}{{\text{v}}_{\text{i}}}\right)+\left(2\rho_{\text{i}}-0.2\right)\left(1-{\text{v}}_{\text{i}}\right)\right), \end{array}$$

where *V*_0_ = 0.02 is the resting blood volume fraction. The biophysical parameters in the simulation were set as ϵ_*i*_ = 0.2, *k*_*i*_ = 0.65, γ_*i*_ = 0.41, τ_*i*_ = 0.98, α_*i*_ = 0.32, and ρ_*i*_ = 0.34. The simulation last for 480 s with a time step of 1 ms, and the first 180 s was removed for stability. The obtained raw BOLD signals were then normalized and sampled every 2 s (TR).

### Avalanche Detection

For the DTI+GH model, the simulated time series were subsequently binarized by assigning the active state to 1 and the other two to 0. Then the raster plot of the activations was divided into many consecutive frames. We calculated the number of activated nodes *N*_*i*_ for frame *i*. In addition, this frame is blank if *N*_*i*_ = 0. If the consecutive frames contain activated nodes, proceeding with blank frame, and ended with blank frame, then the activities in these consecutive frames is defined as an avalanche. The number of total activated nodes in this avalanche is defined as its size. The frame length of DTI+GH model was set to two iteration steps so to obtain avalanche size distribution with power law distribution of −1.5 (Beggs and Plenz, 2003).

For the DTI+EI model, the detection of avalanche in each region is the same as before, except that the activation of nodes is replaced with the firing events of the neurons. The frame length is chosen to be 2 ms so as to produce power law avalanche distributions with exponents closest to −1.5.

## Data Availability Statement

The raw data supporting the conclusions of this article will be made available by the authors, without undue reservation.

## Ethics Statement

The studies involving human participants were reviewed and approved by Second Hospital of Lanzhou University. Written informed consent to participate in this study was provided by the participants' legal guardian/next of kin.

## Author's Note

The hypothesis that the brain might operate at or near-phase transitions because criticality facilitates information processing capabilities and health. This hypothesis was strongly driven by theoretical concepts and supported by many experimental studies. Recent structure-dynamics-function modeling studies combining the structural and functional imaging data at whole brain level demonstrated the functional connectivity (FC) emerges from structural connectivity when the brain dynamics is poised at the criticality. It is therefore conjectured that criticality may facilitate the optimal organization of FC networks, usually characterized by “small worldness” which are corrupted in disordered brains. There are several arguments for this conjecture: First, criticality has been argued to optimize the neural systems for computation, whereas the “small worldness” FC network has been considered an efficient way for inter-regional communication in brains. Second, it has been shown in experiments and simulations that a proper excitation-inhibition (*E*/*I*) balance is required to maintain critical dynamics in cortical networks. Accordingly, *E*/*I* imbalances have been implicated in various brain disorders, such as autism, schizophrenia, etc. In this study, we demonstrated that the FC network organization is optimized by critical dynamics by maximizing the cluster coefficient while minimizing the characteristic path length, so to yield maximal global and local efficiency in information transmission. We also demonstrated with whole brain model that the local *E*/*I* ratio can be optimized to produce critical dynamics in the system, thereby yielding optimal organization of FC networks at the whole brain level.

## Author Contributions

LY and JF designed research. LY, XZ, NM, BS, ZW, and GL performed research. LY, JF, LL, and ZW wrote the paper. All authors reviewed the manuscript.

## Conflict of Interest

The authors declare that the research was conducted in the absence of any commercial or financial relationships that could be construed as a potential conflict of interest.
