# Supplementary material for: Optimal Organization of Functional Connectivity Networks for Segregation and Integration With Large-Scale Critical Dynamics in Human Brains
Source: Front Comput Neurosci. 2021 Mar 31;15:641335. doi: 10.3389/fncom.2021.641335 (PMC8044315; doi:10.3389/fncom.2021.641335)
Supplement: Supplementary file 1 [file Data_Sheet_1.DOCX]

**Supplementary Materials**

**
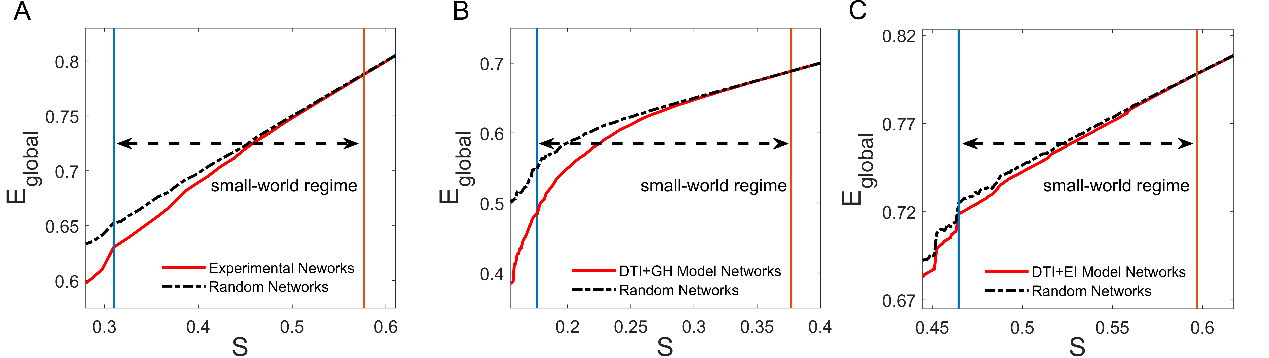
**

**Fig.S1 Global efficiency for the brain FC networks (A) and simulated FC networks based on DTI+GH model(B), as well as DTI+EI model (C) as a function of sparsity compared with random network.** The brain networks (red line in (A)) and simulated networks (red line in (B) and (C)) have lower efficiency than the random networks (black dashed line). The small-world regime is conservatively defined as the range of sparsity for which the global efficiency curve of the networks is lower than the global efficiency curve for the random networks.


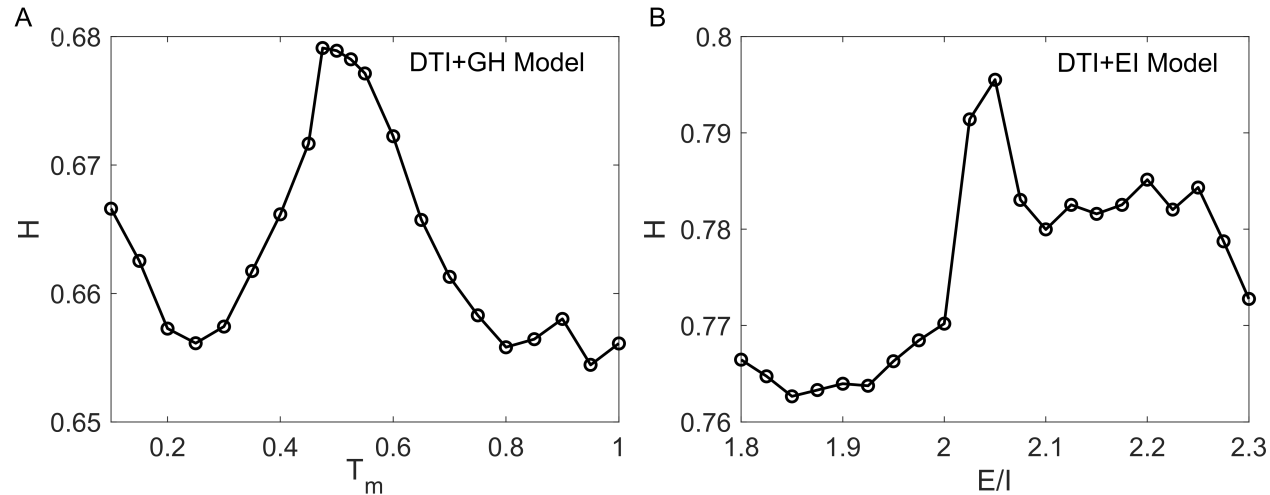


**Figure S2 The dependence of Hurst exponent on the excitation threshold**$\mathbf{T}_{\mathbf{m}}$ **in DTI+GH model (A) and the excitation-inhibition ratio E/I in DTI+EI model (B).** For DTI+GH model, the whole brain Hurst exponent was calculated from the averaged BOLD time series of 200 time points by sampling the raw BOLD signals every 140 iteration steps. For DTI+EI model, the Hurst exponent was calculated by averaging the Hurst exponents of each brain region with a sampling rate of 0.5 Hz and a total time length of 300s.


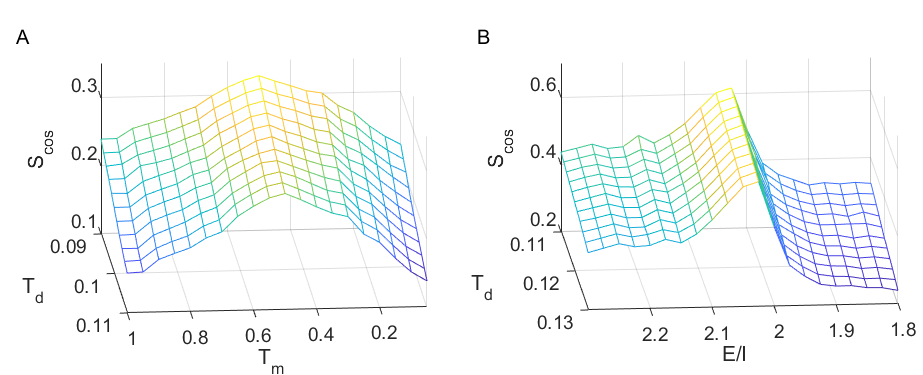


**Figure S3 The dependence of the cosine similarity** $S_{cos}$ **between simulated and experimental FC network on the excitation threshold**$\mathbf{T}_{\mathbf{m}}$ **in DTI+GH model (A) and the excitation-inhibition ratio E/I in DTI+EI model (B)**. To evaluate the similarity between simulated and experimental FC networks, the two kinds of networks were converted into two vectors, and the cosine similarity $S_{cos}$ between these two vectors was calculated. The experimental FC network was calculated by averaging the absolute FC matrix across all subjects.
